# pEGASUS-HPC stent pusher assisted catheterization (PAC) technique in Y-stent-assisted coiling of unruptured wide-necked cerebral aneurysms

**DOI:** 10.1007/s00234-025-03838-0

**Published:** 2025-11-15

**Authors:** Mohammad Almohammad, Bayan Alhaj Moustafa, Ali Khanafer, Mete Dadak, Christopher Nimsky, Alexander Grote, Mariana Gurschi, Abdallah Aburub, Julia Korthäuer, Stephan Felber, Zakarya Ali, Hans Henkes, André Kemmling

**Affiliations:** 1 Department of Neuroradiology, Philipps University of Marburg, Marburg, Germany; 2 Department of Neuroradiology, University Hospital of Giessen and Marburg, Campus Marburg, Marburg, Germany; 3 Department of Neuroradiology, Klinikum Stuttgart, Stuttgart, Germany; 4 Department of Radiology, St. Vincenz Hospital Paderborn, Paderborn, Germany; 5 Department of Neurosurgery, Philipps University of Marburg, Marburg, Germany; 6 Department of Neurosurgery, University Hospital of Giessen and Marburg, Marburg, Germany; 7 Institute for Diagnostic and Interventional Radiology and Neuroradiology, Stiftungsklinikum Mittelrhein, Koblenz, Germany; 8 Central Institute for Diagnostic and Interventional Radiology, Neuroradiology, and Nuclear Medicine, Sana Klinikum Offenbach, Offenbach, Germany

**Keywords:** Stent, Pusher, pEGASUS HPC, Coiling, Catheterization

## Abstract

**Objectives:**

To evaluate the safety and feasibility of the pusher-assisted catheterization (PAC) technique using the pEGASUS-HPC stent pusher instead of a microwire for accessing unruptured wide-necked cerebral aneurysms during Y-stent-assisted coiling.

**Methods:**

In this multicenter retrospective study (July 2021– June 2025), 48 unruptured wide-necked cerebral aneurysms underwent Y-stent-assisted coiling using pEGASUS HPC stents. Based on the catheterization technique, cases were assigned to either the microwire-assisted catheterization (MAC, *n* = 23) or the stent pusher-assisted catheterization (PAC, *n* = 25) group. Clinical and procedural data were analyzed to compare safety and efficacy, focusing on success rates, required catheterization time, complications, and adverse events.

**Results:**

The cohort had a mean age of 62.6 ± 9.8 years, with 64.3% of patients being female. In the MAC group, aneurysm catheterization was successful in all 23 cases (100%), with a mean catheterization time of 5.31 ± 1.2 min. In contrast, the PAC group achieved successful catheterization in 88% of cases (22/25), with a markedly reduced mean catheterization time of 0.82 ± 0.27 min—approximately 6.5 times faster than the conventional MAC technique (*p* < 0.001). Importantly, no procedure-related complications, such as perforations or dissections, were observed in either group.

**Conclusion:**

In this multicenter retrospective feasibility and safety analysis, PAC appeared to enable faster aneurysm access during Y-stent-assisted coiling without an increase in intraprocedural complications. As clinical outcomes were not assessed, these findings should be regarded as technical proof-of-concept and require confirmation in prospective, outcome-driven studies.

## Introduction

In stent-assisted coiling (SAC), two commonly used techniques exist for positioning the tip of the coiling microcatheter within the aneurysm sac, depending on the procedural strategy. The first, known as the jailing technique, involves placing the coiling microcatheter into the aneurysm sac before deploying a stent via a second microcatheter in the parent vessel, thereby trapping (or “jailing”) the coiling catheter between the stent and the vessel wall. The second approach entails first deploying the stent, followed by navigating the coiling microcatheter through the stent struts into the aneurysm sac [1, 2]. In the present study, the latter technique was applied in all cases.

Among the various advanced SAC techniques, Y-stenting has gained particular relevance for bifurcation aneurysms, where conventional strategies often fall short. By creating a Y-shaped scaffold at the vessel bifurcation, this approach enables secure coil placement and effective neck coverage in anatomically challenging cases [3].

Three primary mechanisms of iatrogenic aneurysm perforation during endovascular treatment have been described: perforation caused by the guidewire, the microcatheter, or the coils [4, 5]. Reported incidence rates range from 2% to 4.4%, with a significantly higher risk observed in small aneurysms—especially those measuring less than 4 mm in maximum diameter [4, 6, 7].

Iatrogenic perforation of cerebral aneurysms during endovascular treatment represents a serious complication, associated with morbidity and mortality rates of up to 39%^8^.

The pEGASUS-HPC stent, developed by Phenox GmbH (Bochum, Germany), is a low-profile, self-expanding, laser-cut stent with an open-cell design. It was specifically engineered to conform to various intracranial vessel anatomies and is intended for the treatment of wide-neck aneurysms, arterial dissections, and intracranial stenosis [8, 9]. Its compatibility with standard 0.0165” inner diameter microcatheters used for coiling procedures eliminates the need for microcatheter exchange, thereby simplifying the intervention. Furthermore, the stent is coated with a hydrophilic polymer (HPC) that reduces thrombogenicity by limiting platelet adhesion to its surface [10, 11].

The pusher-assisted catheterization (PAC) technique using the pEGASUS-HPC stent pusher was first described by Alhaj Moustafa et al. in a multicenter retrospective study investigating its use in conventional stent-assisted coiling (SAC) of non-ruptured cerebral aneurysms [12]. Their findings demonstrated high catheterization success rates, significantly reduced procedure time compared to standard microwire-assisted techniques, and a favorable safety profile without procedure-related complications. Building upon these promising results, the present study applies the PAC technique specifically in the context of Y-stent-assisted coiling of wide-necked aneurysms—an anatomically and technically more demanding setting. Our aim was to evaluate whether the advantages of PAC, previously demonstrated in straightforward anatomical settings, also apply to bifurcation aneurysms requiring dual-stent constructs in a Y-configuration.

This study aimed to evaluate the feasibility and technical performance of PAC during Y-stent-assisted coiling, with two predefined procedural endpoints: aneurysm catheterization success and catheterization time. Clinical outcomes, radiation exposure, and cost-related aspects were not assessed, and the present analysis should therefore be regarded as a technical feasibility and safety study.

## Methods

### Study design

A retrospective, sequential analysis across four neurovascular centers, including all consecutive cases treated between June 2021 and June 2025. The first 23 consecutive cases were treated using conventional microwire-assisted catheterization (MAC), which represented the standard approach at that time. Following the introduction of the PAC technique, the subsequent 25 consecutive cases were performed using PAC. Thus, group allocation followed a chronological sequence rather than operator discretion or anatomical considerations, minimizing the risk of selection bias. Nevertheless, the analysis remains observational and hypothesis-generating.

### Study participants

All patients with intracranial wide-necked aneurysms treated by Y-stent-assisted coiling (Y-SAC) using the pEGASUS-HPC stent were included in this study. Wide-necked aneurysms were defined as those with a neck diameter ≥ 4 mm or a dome-to-neck ratio < 2, based on previously established criteria in the literature [13]. Only electively treated, unruptured aneurysms were eligible for inclusion. Patients with acutely ruptured aneurysms were excluded. No additional exclusion criteria were applied and no exclusions were made for other reasons.

### Endovascular procedures

**Fig. 1:**
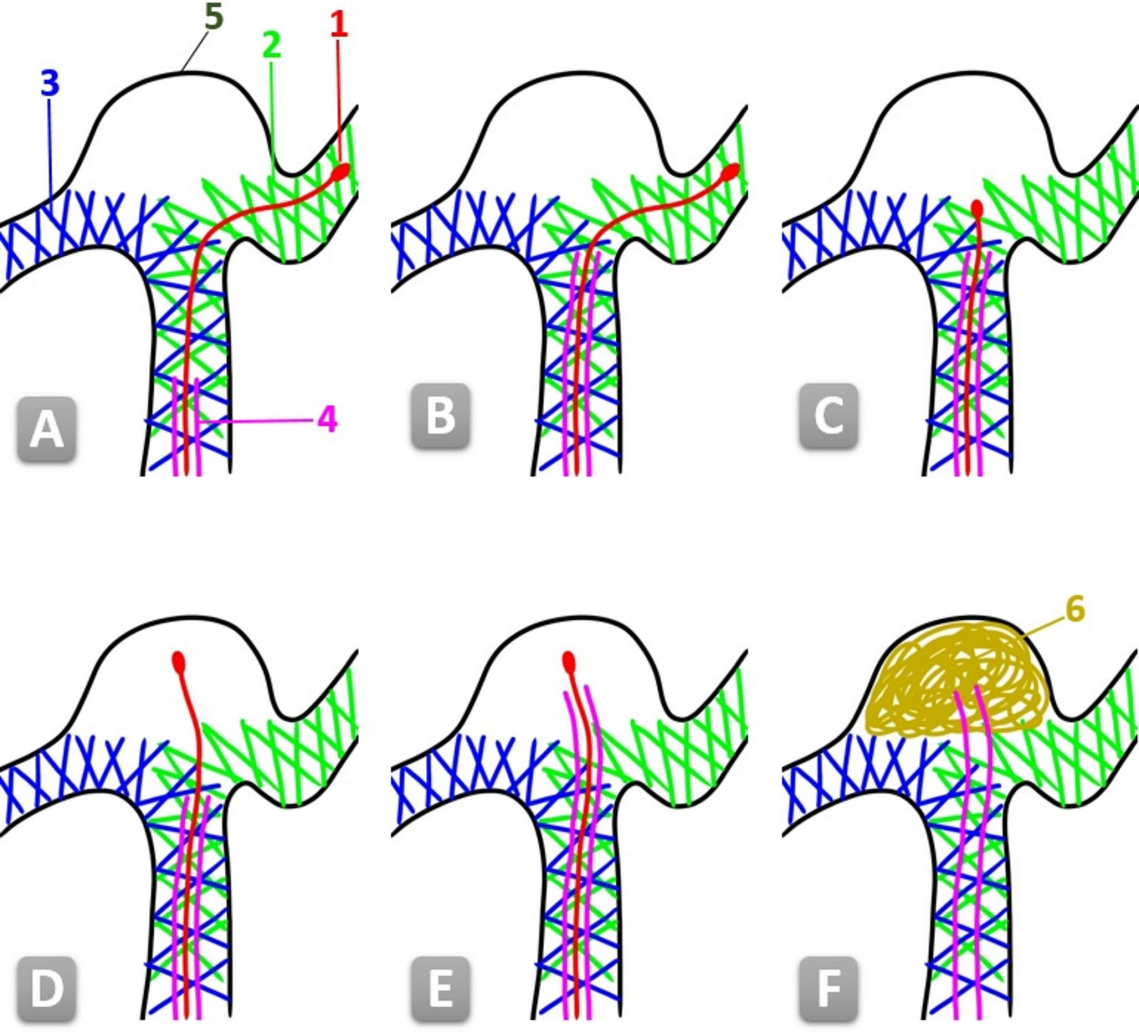
A technical schematic illustrating of the pEGASUS stent pusher-assisted catheterization (PAC) technique for Y-stent-assisted coiling of a bifurcation aneurysm. 1- Stent pusher tip. 2- Second deployed pEGASUS HPC stent. 3- First deployed pEGASUS HPC stent. 4- Microcatheter used for stent delivery and subsequent coiling. 5- The sac of the aneurysm. 6- Coils (A) Anatomy after placement of two pEGASUS HPC stents in a Y-configuration across the aneurysm neck (B) Advancement of the microcatheter toward the level of the neck of the aneurysm (C) Retraction of the stent pusher tip to the aneurysm level (D) Advancement of the stent pusher tip through the stent struts into the aneurysm sac (E) Navigation of the microcatheter into the aneurysm, using the pusher as a rail (F) Withdrawal of the stent pusher and subsequent coil embolization of the aneurysm

All procedures were performed using the pEGASUS-HPC stent system (Phenox GmbH, Bochum, Germany), with two stents deployed in a Y-configuration across the aneurysm neck to reconstruct the bifurcation within the parent vessels. The microcatheter employed for stent delivery was retained for subsequent coil placement, thereby eliminating the need for catheter exchange [12]. Catheterization of the aneurysm sac was achieved by advancing the microcatheter through the cells of the implanted stents. Patients were categorized into two groups according to the catheterization technique used: in the MAC group (n = 23), navigation was facilitated using a Synchro^®^ 0.014” × 215 cm microwire (Stryker^®^); in the PAC group (*n* = 25), the pEGASUS stent pusher itself served as a guiding element for advancing the microcatheter into the aneurysm sac (Fig. 1).

### Data collection

All data were collected in anonymized form and encompassed patient-related variables (age, sex), detailed aneurysm characteristics (including size, neck width, anatomical location, and number of aneurysms per patient), as well as procedural information. The latter included the specific catheterization technique used, duration of catheterization, type of navigation method (microwire or stent pusher), and the stent and coil systems applied. Information on peri-procedural antiplatelet management—such as drug regimen, treatment duration, and response testing—was also recorded. Furthermore, intraoperative complications (e.g., aneurysm perforation, vasospasm, and arterial dissection) and the technical success of aneurysm access were systematically documented.

### Procedural endpoints

The main procedural outcomes assessed were the success of aneurysm catheterization and the duration required for catheter navigation. Successful catheterization was defined as the ability to advance the coiling microcatheter through the stent cells with accurate placement of its tip within the aneurysm sac. Catheterization time was measured from the moment of stent deployment to the point at which the microcatheter was correctly positioned for coil delivery.

### Safety endpoints

Safety assessment focused on the occurrence of intraprocedural complications. Aneurysm perforation was defined as the unintended penetration of the aneurysm wall by the microwire, microcatheter, or embolization coil, resulting in contrast extravasation visible on angiography and indicating subarachnoid leakage. Prompt detection of such events is essential to reduce the risk of serious morbidity and mortality [14, 15]. Accordingly, as part of the standardized safety protocol, a low-volume contrast injection via the intermediate catheter was routinely performed immediately after aneurysm catheterization. Arterial dissection was characterized by an intimal tear leading to intramural hematoma formation and separation of the vessel wall layers, typically presenting with angiographic signs such as a double-lumen configuration, string sign, or intimal flap [16–18]. Advanced imaging techniques, such as diffusion-weighted imaging (DWI), are currently under investigation as a tool for improving the early detection of such dissections [19]. Temporary vasospasm was defined as a transient, angiographically confirmed narrowing of the vessel lumen due to vascular smooth muscle contraction [20]. Other potential complications unrelated to aneurysm access—such as air embolism, thromboembolic events, or distal vessel occlusion—were also systematically evaluated, but none occurred in the present cohort.

### Antiplatelet regimen

All patients were premedicated with dual antiplatelet therapy, comprising prasugrel (10 mg once daily) and acetylsalicylic acid (100 mg once daily), starting five days before the scheduled endovascular intervention. On the day of treatment, platelet function testing was performed using the VerifyNow^®^ system (Werfen) to confirm therapeutic efficacy. No cases of inadequate response were detected; all patients exhibited sufficient platelet inhibition.

### Statistical analysis

Data processing and analysis were conducted using SPSS software (IBM, Version 25, Windows). The distribution of continuous variables was evaluated using the Shapiro–Wilk test. Descriptive statistics are reported as mean ± standard deviation (SD) for normally distributed data, or as median with interquartile range (IQR) for non-normally distributed variables. Categorical data are presented as absolute frequencies and percentages. Group comparisons for continuous variables were performed using the independent Student’s t-test or the Mann–Whitney U test, depending on data normality. Associations between categorical variables were assessed using the Chi-square test or Fisher’s exact test, where applicable. A p-value below 0.05 (two-tailed) was considered indicative of statistical significance.

## Results

###  Demographic and clinical characteristics

Between June 2021 and June 2025, a total of 42 patients with 48 incidental, unruptured intracranial aneurysms underwent elective Y-stent-assisted coiling (Y-SAC) using two pEGASUS-HPC stents per case. The mean patient age was 62.6 ± 9.8 years, with 64.3% of patients being female (*n* = 27). The aneurysms had an average neck width of 4.6 ± 1.7 mm, a mean sac width of 5.9 ± 2.9 mm, and a mean sac depth of 5.3 ± 3.1 mm. The mean dome-to-neck ratio was 1.3 ± 0.7. The most frequent aneurysm location was the anterior communicating artery (AComA, *n* = 16; 33.3%), followed by the basilar artery (BA, *n* = 14; 29.2%) and the middle cerebral artery (MCA, *n* = 12; 25%). Aneurysms in the anterior circulation (*n* = 34; 70.8%) were more common than those in the posterior circulation. With regard to laterality, 20.8% (*n* = 10) of aneurysms were located on the left side, 16.7% (*n* = 8) on the right, and 62.5% (*n* = 30) were located midline in the AComA or BA. All aneurysms were saccular and had not been previously treated (Table 1).

**Table 1:** Demographic, clinical, and imaging characteristics. AcomA, anterior communicating artery. BA, Basilar artery. ICA, internal carotid artery. MCA, middle cerebral artery. PcomA, posterior communicating artery. MAC, microwire-assisted catheterization. PAC, pusher-assisted catheterization. SD, standard deviation

Parameters		*N* (%)/Mean ± SD		
Group		All	MAC group	PAC group
Patient characteristics (*n* = 42)				
Number of Patients		42 (100%)	23 (54.8%)	19 (45.2%)
Age, years		62.6 ± 9.8	63.4 ± 10.2	61.9 ± 9.5
Gender, female		27 (64.3%)	14 (60.9%)	13 (68.4%)
Aneurysm characteristics (*n* = 48)				
Number of aneurysms		48 (100%)	23 (47.9%)	25 (52.1%)
Aneurysm Location	AcomA	16 (33.3%)	8 (34.8%)	8 (32%)
	BA	14 (29.2%)	6 (26.1%)	8 (32%)
	MCA	12 (25%)	6 (26.1%)	6 (24%)
	ICA	3 (6.2%)	1 (4.3%)	2 (8%)
	PcomA	3 (6.2%)	2 (8.7%)	1 (4%)
Anterior vs. Posterior	Anterior	34 (70.8%)	16 (69.6%)	18 (72%)
Aneurysm Laterality	Left	10 (20.8%)	7 (30.4%)	7 (28%)
	Right	8 (16.7%)	5 (21.7%)	5 (20%)
	Midline	30 (62.5%)	4 (17.4%)	4 (16%)
Aneurysm Neck-Width		4.6 ± 1.7	4.7 ± 1.6	4.5 ± 1.8
Aneurysm Sack-Width		5.9 ± 2.9	6.0 ± 2.8	5.8 ± 2.9
Aneurysm Sack-depth		5.3 ± 3.1	5.5 ± 3.2	5.1 ± 3.0
Dome to neck Ratio		1.3 ± 0.7	1.2 ± 0.6	1.3 ± 0.7

### Procedural endpoints

In the MAC group, aneurysm catheterization was successful in all 23 cases (100%), with a mean catheterization time of 319 ± 72 s (5.31 ± 1.2 min). In the PAC group, catheterization was successful in 22 out of 25 cases (88%). The mean catheterization time was significantly shorter at 49 ± 16 s (0.82 ± 0.27 min). While the difference in catheterization success did not reach statistical significance (*p* = 0.235, Fisher’s exact test), the reduction in catheterization time in the PAC group was statistically significant (*p* < 0.001, unpaired two-tailed t-test). PAC was unsuccessful in three cases (12%), all involving aneurysms with tangential or side-wall take-off that prevented coaxial alignment of the pusher with the aneurysm neck. In each instance, successful catheterization was subsequently achieved with MAC. The two groups therefore reflect sequential, consecutive case series (MAC first, PAC subsequently after its introduction), without selective allocation. (Table 2).

**Table 2:** Catheterization success, catheterization time, and procedural complications. MAC, microwire-assisted catheterization. PAC, pusher-assisted catheterization. SD, standard deviation

Parameters	*N* (%)/Mean ± SD	
	MAC group (*n* = 23)	PAC group (*n* = 25)
Catheterization success, n (%)	23 (100%)	22 (95%)
Catheterization time, seconds (mean ± SD)	319 ± 72	49 ± 16
Catheterization time, minutes (mean ± SD)	5.31 ± 1.2	0.82 ± 0.27
Perforations, n (%)	0 (0%)	0 (0%)
Vasospasms, n (%)	3 (13%)	4 (16%)
Dissections, n (%)	0 (0%)	0 (0%)

### Procedural complications

No intraprocedural aneurysm perforations or dissections were observed in either group. Temporary vasospasm occurred in 3 cases (13%) in the MAC group—affecting the vertebral artery (*n* = 1) and internal carotid artery (*n* = 2)—and in 4 cases (16%) in the PAC group (vertebral artery: *n* = 2; internal carotid artery: *n* = 2). In all instances, vasospasm developed during navigation of the triaxial system through tortuous vessel anatomy. Treatment consisted of intra-arterial infusion of 2 mg nimodipine diluted in 1 L of saline via the guiding catheter, resulting in full angiographic resolution during the procedure. No clinical sequelae were observed, and no further periprocedural complications occurred in either group (Table 2).

## Discussion

This multicenter retrospective cohort study evaluated the safety and efficacy of a novel catheterization approach—pusher-assisted catheterization (PAC)—for Y-stent-assisted coiling (Y-SAC) of wide-necked, unruptured cerebral aneurysms. In contrast to the conventional microwire-assisted catheterization (MAC) technique, PAC employs the pEGASUS-HPC stent pusher to guide the coiling microcatheter through the stent struts into the aneurysm sac. To our knowledge, this is the first systematic clinical investigation of this technique specifically in the challenging context of dual-stent Y-constructs at intracranial bifurcations.

The PAC technique was first introduced by Alhaj Moustafa et al. in the setting of pEGASUS-HPC stent assisted coiling (SAC), where it showed high efficacy and a favorable safety profile [12]. Building upon this prior work, the present study marks a significant advancement by applying the pEGASUS-HPC PAC technique to the technically more demanding setting of wide-necked bifurcation aneurysms, where dual-stent Y-configurations are necessary for adequate neck coverage.

The overall safety profile of both techniques (MAC and PAC) was favorable, with no intraprocedural aneurysm perforations or dissections in either group. Temporary vasospasm occurred in 13% of MAC and 16% of PAC cases, typically during triaxial system navigation. All vasospasms resolved completely after intra-arterial nimodipine administration, and no clinical sequelae were observed. These findings suggest that PAC does not increase the risk of procedural complications, even in the technically demanding setting of Y-SAC.

**Fig. 2:**
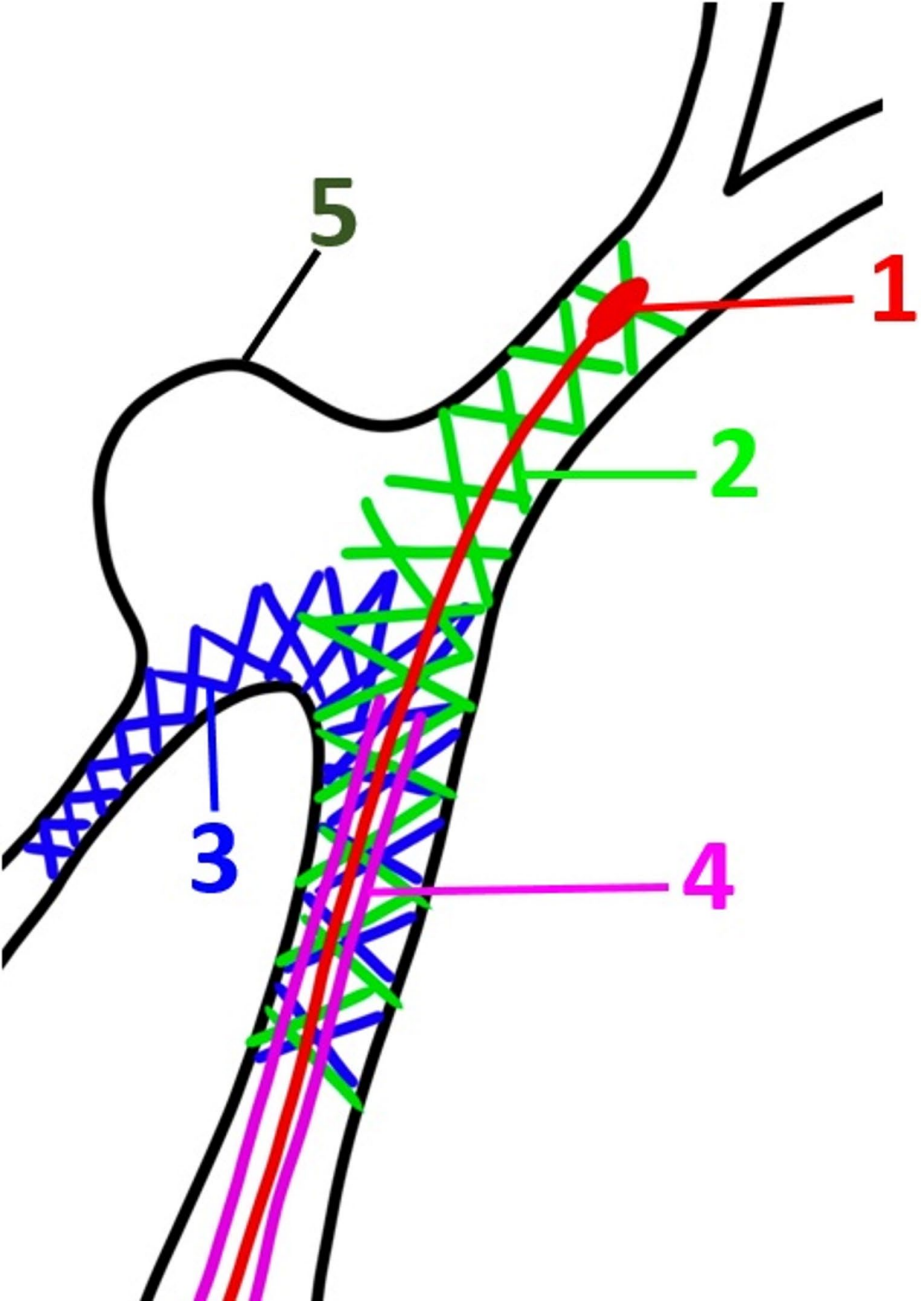
A technical schematic illustrating the anatomical limitation of the PAC technique. In this example, the aneurysm arises tangentially from the parent vessel, preventing coaxial alignment of the stent pusher with the aneurysm neck and resulting in unsuccessful catheterization. 1- Stent pusher tip. 2- Second deployed pEGASUS HPC stent. 3- First deployed pEGASUS HPC stent. 4- Microcatheter used for stent delivery and intended for coiling. 5- The sac of the aneurysm

The catheterization success rate was slightly lower in the PAC group (88%) compared to 100% in the MAC group; however, this difference did not reach statistical significance (*p* = 0.235). Importantly, all failed PAC attempts were successfully salvaged by switching to MAC, demonstrating that the technique can be safely integrated into existing workflows. As observed in the original description of PAC for SAC, catheterization failures were primarily associated with tangential aneurysm configurations, which may impair the passive alignment of the pusher tip with the aneurysm orifice (Fig. 2).

The most striking advantage of PAC was its marked reduction in catheterization time. With an average access time of 0.82 ± 0.27 min, PAC was approximately 6.5 times faster than MAC (5.31 ± 1.2 min, *p* < 0.001). This result is consistent with previous findings from the single-stent SAC and highlights the applicability of PAC even in Y-stenting procedures, where vascular anatomy and dual-stent configurations are more complex and catheterization inherently more demanding. The significant time savings associated with PAC may translate into reduced fluoroscopy exposure, lower contrast volume, and decreased operator fatigue—factors that collectively improve procedural safety and efficiency.

Beyond workflow considerations, PAC may provide several patient-centered advantages. The marked reduction in catheterization time shortens the unprotected interval between stent deployment and aneurysm catheterization, potentially lowering rupture risk in complex Y-SAC procedures. By avoiding microwire manipulation within or near the sac, PAC reduces intraluminal handling, which may in theory diminish the risk of iatrogenic vessel injury. Moreover, eliminating the need for a microwire exchange may reduce the chance of device-related complications such as air embolism. Finally, shorter catheterization time may contribute to reduced fluoroscopy exposure and contrast usage. While these benefits remain hypothetical, they provide a rationale for further prospective studies explicitly designed to evaluate patient-level outcomes.

Overall, the PAC technique appears particularly effective in favorable aneurysm orientations, where the sac aligns coaxially with the parent vessel, allowing smooth advancement of the microcatheter over the pusher. In anatomically complex or tangential configurations, MAC remains a reliable and immediately available fallback strategy.

### Limitations

This study has several limitations. Its retrospective nature introduces potential bias. Because allocation followed a chronological sequence (MAC in the first 23 consecutive cases, PAC in the subsequent 25 cases after its introduction), operator-driven selection bias was minimized. Nevertheless, the absence of randomization means that the comparison remains observational, and only prospective or randomized studies can provide a robust benchmark. Endpoints were restricted to procedural feasibility and access time, without evaluation of diagnostic yield, therapeutic targeting, avoidance of unnecessary procedures, or clinical outcomes; thus, the results represent a technical proof-of-concept rather than evidence of direct patient benefit. Although the sample size exceeds that of many comparable Y-stenting series, it remains relatively modest. The exclusive use of the pEGASUS-HPC stent limits generalizability, as performance may differ with other platforms. Long-term clinical and angiographic follow-up was not included and should be addressed in future prospective trials. Radiation dose, fluoroscopy time, and contrast volume were not recorded, and therefore any potential impact of PAC on these safety parameters could not be determined.

Ethics Considerations: The PAC technique was evaluated as a modification of standard endovascular practice using CE-marked devices. The purpose was to assess technical feasibility and procedural safety, not to demonstrate economic or administrative benefit. No patient-level outcomes were measured, and no claims of clinical advantage are made. Potential benefits, such as reduced manipulation within the aneurysm or shorter catheterization time, remain hypothetical and require confirmation in prospective outcome-driven studies before implementation beyond individual operator preference can be ethically justified.

## Conclusion

In summary, the PAC technique using the pEGASUS-HPC stent appears to be a feasible and time-saving adjunct for aneurysm access during Y-stent-assisted coiling of unruptured wide-necked bifurcation aneurysms. While the approach reduced catheterization time compared with conventional microwire navigation, it did not demonstrate improved clinical outcomes in this retrospective sequential series. Given the modest sample size, the non-randomized design, and the absence of radiation, contrast, or follow-up data, the clinical relevance of the observed time reduction remains uncertain. These results should therefore be regarded as hypothesis-generating. Prospective studies with larger cohorts, systematic collection of fluoroscopy and contrast metrics, and incorporation of patient-level outcomes are needed to determine whether PAC translates into measurable clinical benefit.

## Data Availability

No datasets were generated or analysed during the current study.

## Abbreviations

ACA: anterior cerebral artery
AComA: anterior communicating artery
BA: basilar artery
DWI: diffusion-weighted imaging
HPC: hydrophilic polymer coating
ICA: internal carotid artery
i.a.: intra-arterial
IRB: institutional review board
IQR: interquartile range
MAC: microwire-assisted catheterization
MCA: middle cerebral artery
PAC: pEGASUS HPC stent pusher-assisted catheterization
PComA: posterior communicating artery
SAC: stent-assisted coiling
SD: standard deviation
VA: vertebral artery
